# Loss of MMP-8 in ductal carcinoma in situ (DCIS)-associated myoepithelial cells contributes to tumour promotion through altered adhesive and proteolytic function

**DOI:** 10.1186/s13058-017-0822-9

**Published:** 2017-03-23

**Authors:** Muge Sarper, Michael D. Allen, Jenny Gomm, Linda Haywood, Julie Decock, Sally Thirkettle, Ahsen Ustaoglu, Shah-Jalal Sarker, John Marshall, Dylan R. Edwards, J. Louise Jones

**Affiliations:** 1 Translational Cancer Discovery Team, CRUK Cancer Therapeutics Unit, Institute of Cancer Research, 15 Cotswold Road, Sutton, Surrey, SM2 5NG UK; 2 Centre for Tumour Biology, Barts Cancer Institute, John Vane Science Centre, Charterhouse Square, Queen Mary University of London, Charterhouse Square, London, EC1M 6BQ UK; 3 Cancer Research Centre, Qatar Biomedical Research Institute, Qatar Foundation, Doha, Qatar; 4 Centre for Experimental Cancer Medicine, Barts Cancer Institute, John Vane Science Centre, Charterhouse Square, Queen Mary University of London, Charterhouse Square, London, EC1M 6BQ UK; 5 School of Biological Sciences, University of East Anglia, Norwich Research Park, Norwich, NR4 7TJ UK

**Keywords:** Ductal carcinoma in situ, Myoepithelial cell, Microenvironment, MMP-8, Adhesion, Hemidesmosomes, Organotypic assays, Invasion

## Abstract

**Background:**

Normal myoepithelial cells (MECs) play an important tumour-suppressor role in the breast but display an altered phenotype in ductal carcinoma in situ (DCIS), gaining tumour-promoter functions. Matrix metalloproteinase-8 (MMP-8) is expressed by normal MECs but is lost in DCIS. This study investigated the function of MMP-8 in MECs and the impact of its loss in DCIS.

**Methods:**

Primary normal and DCIS-associated MECs, and normal (N-1089) and DCIS-modified myoepithelial (β6-1089) cell lines, were used to assess MMP-8 expression and function. β6-1089 lacking MMP-8 were transfected with MMP-8 WT and catalytically inactive MMP-8 EA, and MMP-8 in N-1089 MEC was knocked down with siRNA. The effect on adhesion and migration to extracellular matrix (ECM), localisation of α6β4 integrin to hemidesmosomes (HD), TGF-β signalling and gelatinase activity was measured. The effect of altering MEC MMP-8 expression on tumour cell invasion was investigated in 2D and 3D organotypic models.

**Results:**

Assessment of primary cells and MEC lines confirmed expression of MMP-8 in normal MEC and its loss in DCIS-MEC. Over-expression of MMP-8 WT but not MMP-8 EA in β6-1089 cells increased adhesion to ECM proteins and reduced migration. Conversely, knock-down of MMP-8 in N-1089 reduced adhesion and increased migration. Expression of MMP-8 WT in β6-1089 led to greater localisation of α6β4 to HD and reduced retraction fibre formation, this being reversed by MMP-8 knock-down in N-1089. Over-expression of MMP-8 WT reduced TGF-β signalling and gelatinolytic activity. MMP-8 knock-down enhanced TGF-β signalling and gelatinolytic activity, which was reversed by blocking MMP-9 by knock-down or an inhibitor. MMP-8 WT but not MMP-8 EA over-expression in β6-1089 reduced breast cancer cell invasion in 2D and 3D invasion assays, while MMP-8 knock-down in N-1089 enhanced cancer cell invasion. Staining of breast cancer cases for MMP-8 revealed a statistically significant loss of MMP-8 expression in DCIS with invasion versus pure DCIS (*p* = 0.001).

**Conclusions:**

These data indicate MMP-8 is a vital component of the myoepithelial tumour-suppressor function. It restores MEC interaction with the matrix, opposes TGF-β signalling and MMP-9 proteolysis, which contributes to inhibition of tumour cell invasion. Assessment of MMP-8 expression may help to determine risk of DCIS progression.

**Electronic supplementary material:**

The online version of this article (doi:10.1186/s13058-017-0822-9) contains supplementary material, which is available to authorized users.

## Background

Many invasive breast cancers (IBC) develop through a pre-invasive stage known as ductal carcinoma in situ (DCIS) where the proliferative neoplastic cells are retained within the breast duct surrounded by an intact myoepithelial cell (MEC) layer lying in contact with basement membrane [1]. DCIS is a non-obligatory precursor of IBC; only around half of untreated cases progress to IBC, when the tumour cells breach the MEC-basement membrane barrier [2, 3]. However, the molecular mechanisms underlying the transition of DCIS to IBC are poorly understood, and identifying those cases that will progress and those that will not remains a major challenge [4, 5].

Molecular profiling studies demonstrate that DCIS tumour cells and their invasive counterparts share a similar signature and DCIS is as genetically advanced as established invasive disease [6, 7]. Therefore, in order to understand the changes that trigger invasion, attention has focused on the role of the microenvironment [8–11]. MECs are a unique component of the microenvironment in the breast. In normal breast ducts, MECs adhere tightly to the basement membrane using prominent hemidesmosome (HD) formation [12]. They show a high level of expression of anti-tumourigenic factors, such as proteinase inhibitors and anti-angiogenic mediators, and in vitro studies have demonstrated broad tumour-suppressor activities of normal primary MEC and MEC lines [13–15]. In DCIS, the MECs become altered, showing changes in gene expression, epigenetics and phenotype [13, 14, 16, 17], though the functional significance of these changes has not been established [9, 18–20].

A previous study has indicated that in normal ducts MECs appear to be the main source of matrix metalloproteinase-8 (MMP-8), which is a major collagenase that cleaves Collagen type- I (Col -I) [21]. Expression of MMP-8 appears to be lost in DCIS-associated MECs [22]. MMPs are a large family of endopeptidases, which have the ability to remodel extracellular matrix (ECM) and are upregulated in many cancers, such that the MMP family is conventionally regarded as consisting of key enzymes that contribute to the process of cancer cell invasion and metastasis [3, 23–25]. However, the failure of broad range MMP inhibitors in anticancer trials suggests an incomplete understanding of the complex function of this family; specifically when and how they act in cancer [3, 23–25]. Moreover; regulatory interactions (compensation or inhibition) between MMP family members construct a complex web through which the expression and activity of MMPs are constantly controlled [25, 26]. One particular example is MMP-9, which is implicated in the acquisition of DCIS-associated phenotype in MECs [27] at the same stage when MECs lose MMP-8 expression. Since it was first reported that MMP-8^-/-^ mice exhibit increased incidence of skin tumours when challenged with chemical carcinogenesis, MMP-8 has been demonstrated to exert a clear tumour-suppressor function [22, 26, 28, 29]. There is a growing body of evidence accumulating that MMP-8 has an anticancer role in breast cancer, malignant melanoma and tongue squamous cell carcinoma, [22, 26, 28, 30–32]. Interestingly, identification of non-structural substrates of MMP-8 [28, 33–37], suggests that the biological function of MMP-8 is much more complicated than just Col-I degradation.

The biological significance of loss of MEC-derived MMP-8 on MEC phenotype and MEC-breast cancer cell crosstalk remains elusive, especially whether MMP-8 may contribute to the tumour-suppressor function of MECs. In this study we employed 2D and 3D in vitro models to recapitulate the DCIS tumour microenvironment in order to investigate how MMP-8 is involved in MEC-breast cancer cell communication, and whether loss of MMP-8 contributes to loss of tumour-suppressor activity and promotes progression to invasive disease. Improving our understanding of the factors that contribute to transition of DCIS to invasion will aid in the future development of predictive signatures to help stratify patient management more appropriately, and avoid the ‘overtreatment’ that has caused such controversy in the breast screening programme.

## Methods

### Immunohistochemistry

Breast tissue samples were obtained from surgical specimens from patients undergoing breast surgery at Barts Health NHS Trust London. The study was performed following patient consent and approval from the local research ethics committee (reference: 05/Q0403/199 and 09/H075/39). Seven were normal breast cases from reduction mammoplasty, nine were cases of DCIS (high, intermediate and low grade) and nine were cases of DCIS with concomitant invasion.

Sections were dewaxed in xylene and antigen retrieved in citrate buffer pH6; followed by incubation with MMP-8 rabbit polyclonal antibody (Atlas, Cambridge, MA, USA, HPA02122, 1:750). Sections subsequently were incubated with goat anti-rabbit biotinylated F(ab’)2 for 30 min, developed using ABC reagent and superDAB (Dako, Glostrup, Denmark) then counterstained with hematoxylin [7].

### Cell lines and cell culture

All breast cancer cell lines were obtained from American Type Culture Collection (ATCC) and verified with STR profiling (LGC Standards, Teddington, UK, tracking number 710081047). MCF-7 and MDA-MB-231 cells were cultured in 10% fetal bovine serum (FBS, PAA Laboratories, Pasching, Austria, A15-14) containing (complete) DMEM (PAA Laboratories, E15-843). SUM159 cells were grown in complete Ham’s F12 (PAA Laboratories, E15-817) with hydrocortisone (Sigma-Aldrich, St. Louis, MO, USA, HO 888, 1 μg/ml) and insulin (Sigma-Aldrich, I9278, 1 μg/ml).

### Generation of myoepithelial cell lines

The h-TERT/SV40 LgT immortalised MEC line was a gift from M. O’Hare and P. Jat, Institute of Neurology, UCL, London. αvβ6 integrin over-expressing and control (normal) MEC line was generated as previously described [27], and termed β6-1089 and N-1089 respectively. These cells were cultured in the presence of hydrocortisone (1 μg/ml), epidermal growth factor (EGF, Sigma-Aldrich, 9644, 10 ng/ml), insulin (1 μg/ml) and puromycin (1 μg/ml, Sigma-Aldrich) in Ham’s F12.

### Isolation of primary myoepithelial and luminal epithelial populations from normal breast and DCIS tissue

Primary cell isolation protocol was adapted from Gomm et al. [38]. In brief, reduction mammoplasty tissue or DCIS tissue was digested overnight at 37 °C with 1 mg/ml collagenase (Sigma-Aldrich, C2674) and hyaluronidase (Sigma-Aldrich, H3506) on a roller-mixer. Following washing and a sedimentation step the organoids were further digested with trypsin/EDTA (PAA Laboratories, L11-004) and DNase (Roche Diagnostics, Basel, Switzerland, 10104159001) then filtered through a 40-μm filter (Becton Dickinson, Franklin Lakes, NJ, USA). Cells were incubated in a 1:1 ratio sequentially with magnetic beads (Dynal Biotech., Milwaukee, WI, USA) coated with common acute lymphoblastic leukemia antigen (CALLA) antibody (AbD Serotech, Oxford, UK, MCA1556) to isolate normal MECs or anti-β4 integrin antibody (EMD Millipore, Billerica, MA, USA, mAB 1964) for DCIS myoepithelial cells. This was followed by epithelial cell adhesion molecule (EpCAM) antibody-coated epithelial-enriched magnetic beads (Invitrogen, Carlsbad, CA, USA, 161.02) to isolate luminal epithelial cells (LECs). Primary fibroblasts were grown from the media produced by the sedimentation step. The purity of these populations has been verified by staining for CK8, CK18, EMA for the luminal epithelial cells and CK14, SMA, CD10, vimentin, p63 for the myoepithelial cells, as described in previous publications [27, 38].

### MMP-8 over-expression

The wild-type MMP-8 (WT) and inactive mutant MMP-8 (EA) coding inserts cloned into pcDNA4 (EcoRI and XhoI restriction sites, Invitrogen, K103002) have been described previously [39]. Empty pcDNA4 vector was used as a control.

β6-1089 cells were plated at a density 7 × 10^4^ cells per well onto 6-well plates 24 hours prior to transfection. β6-1089 cells were transfected with 1 μg DNA per well with Genejuice reagent (Novagen, Merck KGaA, Darmstadt, Germany, 70967) according to the manufacturer’s instructions for 5 hours at 37 °C. All functional experiments were performed 24 hours after transfection. For generation of conditioned media (CM), complete media was replaced with serum-free media (SFM) and harvested after 24 hours, followed by centrifugation at 1200 rpm for 3 minutes to remove cell debris and storage at -80 °C.

### MMP-8 knock-down

N-1089 cells were seeded onto six-well plates at a density 7 × 10^4^ cells per well and incubated for 24 hours at 37 °C, after which media was replenished and N-1089 cells were transfected with 5nM pooled MMP-8 siRNA (Thermo Fisher Scientific, Waltham, MA, USA, MU-005969-00-0020) or Luciferase GL3 Duplex control siRNA (siLUC, Thermo Fisher Scientific, D-001400-01-20) [40] using interferin transfection reagent (Polyplus tranfection, Peqlab Biotechnologie GmbH, Erlangen, Germany, 409-01) according to the manufacturer’s instructions. All functional assays were carried out 96 hours after transfection. To generate CM, cells were serum starved for 24 hours and media was harvested as described above.

### Reverse transcription and q-PCR

RNA was extracted with RNeasy kit (Qiagen, Hilden, Germany, 74104) and reverse transcribed with Superscript II (Invitrogen, 11064-014). One hundred nanograms of cDNA were used per reaction.

All primers made up at 100 mM stock concentration and used at 0.4 mM final concentration. Reactions were performed using Immomix Red Master Mix (Bioline, London, UK, Bio 25-022) in a total reaction volume of 25 μl. For nested PCR, 2 μl PCR product was used as a template for second-round PCR. The products were separated on a 1% agarose (Invitrogen, 16500-500) gel for 45 min at 100v and visualised under UV light (AutoChemi System, UVP, Cambridge, UK).

Q-PCR was carried out using SYBR Green (Applied Biosystems, Foster City, CA, USA, 4367659) chemistry. One hundred nanograms of cDNA input was used per reaction. Primers were used at 0.3 mM final concentration in 10 μl total PCR volume for one well of a 96-well plate. Reactions were carried out on a Step One Plus instrument (Applied Biosystems).

Primer sequences - MMP-8 forward: 5′-GCCGAAGAAACATGGACCAAC-3′, MMP-8 nested forward: 5′- ACTCCTCTGACCCTGGTGCC-3′, MMP-8 reverse: 5′- TGAGGATGCCTTCTCCAGAAG-3′, αvβ6 forward: 5′- GAAGGAATGATCACGTACAAG-3′, αvβ6 reverse: 5′- AGCAGGGAGTCTTCACAGGT-3′, 18 s forward: 5′-CACGGGAAACCTCACCCGGC-3′, 18 s reverse: 5′- AGCAGGGAGTCTTCACAGGT-3′.

### Western blotting

Cells were lysed with radioimmunoprecipitation (RIPA) buffer. CM from N-1089 and β6-1089 cells was concentrated 20× using centrifugal units (EMD Millipore, 4FC 800324) with 3 K molecular weight cutoff (MWCO) at 4000 g for 45 minutes at 4 °C. Samples were boiled in reducing (β-mercaptoethanol containing) laemmli buffer for 5 minutes at 95 °C, then separated with SDS (National Diagnostics, Atlanta, GA, USA, EC874) acrylamide (National Diagnostics, EC890) gel for 90 minutes at 125 V at room temperature. Recombinant human MMP-8 western blot standard (R&D Systems, Minneapolis, MN, USA, WBC017) was loaded as positive control. The gel was transferred to a nitrocellulose membrane (Hybon C extra, RPN203E) for 90 minutes at 30 V at room temperature and Western blotting was performed as previously described [27].

Primary antibodies used included mouse anti-human MMP-8 antibody (R&D Systems, mAb 908), SMAD2 (Cell Signaling Technology, Danvers, MA, USA, 3122), pSMAD2 (Cell Signaling Technology, 3101). Protein loading was confirmed with HSC70 (Santa Cruz Biotechnology, Dallas, TX, USA, sc-7298) or actin (Santa Cruz Biotechnology, sc-1615) as loading controls. For MMP-8 over-expressed samples, the membrane was probed with anti-V5 antibody (Invitrogen, R960-25). Depending on the primary antibody species, horseradish peroxidase (HRP)-conjugated mouse secondary (Dako, P0447), rabbit secondary (Dako, P0448) or goat secondary (Dako; P0160) antibody was used. The membrane was developed with ECL (GE Healthcare, Chicago, IL, USA, RPN 2106) and densitometric quantification analysis was performed with Image J software (National Institutes of Health, Bethesda, MD, USA).

### Invasion assays

Boyden chamber transwells (Corning, Corning, NY, USA, 3422, pore size: 8 μm) coated with 70 μl diluted Matrigel (1 volume Matrigel: 2 volumes ice-cold serum-free media) were used to measure in vitro invasive capacity, as previously described [27]. 2 × 10^4^ modified β6-1089 or N-1089 cells were plated in serum-free media in the lower chamber. 3 × 10^4^ MCF-7, MDA-MB-231or SUM159 breast cancer cells were seeded onto the Matrigel-coated insert. Invasion assays were carried out at 37 °C over 24 hours, or 48 hours for MCF-7 cells. Invaded cells were harvested with 10× trypsin/EDTA (PAA Laboratories, L11-003) from the underside of the transwell and counted with a CASY counter (Schärfe System, Reutlingen, Germany).

### Viability assay

To analyse MEC viability, 3 × 10^4^ β6-1089 or N-1089 cells were seeded onto a 24-well plate and incubated for 24 hours at 37 °C after which the media was replenished and MTS reagent (Promega, Madison, WI, USA, cell titer 96 Aqueous, G5421) was added (5:1 media:MTS v/v ratio) then incubated for 1 hour at 37 °C. The MTS containing mixed media was then collected and placed on a 96-well plate and read at 495 nm (Tecan, Männedorf, Switzerland, infiniteF50).

To analyse breast cancer cell viability 3 × 10^4^ MCF-7, MDA-MB-231 or SUM159 cells were incubated with CM collected from modified β6-1089 or N-1089 cells for 24 hours. Viability was quantified using MTS reagent as described above.

### Adhesion assays

Non-tissue culture-treated 96-well plates were coated with 100 μl of the following matrices in triplicate at given concentrations: Fibronectin at 1 μg/ml, rat tail Collagen-I (BD Biosciences, Franklin Lakes, NJ, USA, 354236) at 0.5 μg/ml, Matrigel at 0.5 μg/ml, Tenascin-C (EMD Millipore, CC 065) at 3 μg/ml, Laminin-I at 10 μg/ml, Collagen-IV at 10 μg/ml and latency-associated peptide (LAP) (Sigma-Aldrich, L3408) at 0.5 μg/ml. Three wells of each plate were coated with 0.1% bovine serum albumin (BSA, PAA Laboratories, K45-001) in phosphate-buffered saline (PBS) (w/v) as a control. Coated plates were incubated for 1 hour at 37 °C after which wells were washed twice with 100 μl PBS per well.

Modified β6-1089 or N-1089 cells were serum starved for 24 hours prior to the experiment. 3 × 10^3^ cells were seeded in 100 μl of serum-free Ham’s F12 per well and allowed to adhere for 1 hour at 37 °C. Then 0.25 μl of Calcein AM (Invitrogen, C1430) cell tracker was added and incubated for 15 minutes at 37 °C after which the plate was washed twice with 100 μl PBS per well, and 100 μl of SFM was added. The plate was read at 490/520 nm on a fluorescent reader (BMG Labtech, Aylesbury, UK, FLUOstar Optima). The adhesion was calculated as the fluorescence value of adherent cells and normalised to that of control cells.

### Migration assays

The underside of Boyden chamber transwells were coated with 100 μl of ECM or 0.1% BSA in PBS as a control, and incubated for 1 hour at room temperature. ECM components and concentrations were as for the adhesion assays. Following incubation the underside of the transwell was washed with 100 μl PBS and placed into 500 μl of SFM containing well of a 24-well plate.

3 × 10^4^ modified β6-1089 or N-1089 cells were added onto the inner chamber in 200 μl serum-free Ham’s F12 and incubated for 8 hours at 37 °C. After that the media in the inner and outer chambers were replaced with 200 μl and 500 μl 10× trypsin EDTA respectively and incubated for 1 hour at 37 °C. The cells were then trypsinised and the migrated cells as well as the number of cells remaining in the inner chamber were counted using a CASY counter. The percentage of migrated cells was calculated using the counts of the upper chamber versus total cell number.

### Organotypic culture

Organotypic gels were constructed using rat tail Collagen-I and Matrigel as previously described [27]. Briefly; for 10 gels, 4.9 ml rat tail Collagen-I was mixed with 2.1 ml Matrigel (7:3 v/v ratio), 1 ml 10× DMEM (Sigma-Aldrich, D2429) and 1 ml FBS. The pH of the solution was neutralised by adding 1 M NaOH (Sigma-Aldrich, S8045) drop-wise until the mixture turned an orange-pink colour. 5 × 10^6^ primary normal breast fibroblasts were suspended in 1 ml complete DMEM and added to the neutralised gel mixture. One millilitre of gel mixture was added to 1 well of a 24-well plate and set for 1 hour at 37 °C. Following this, complete DMEM was added drop-wise on top of the gels and equilibrated overnight at 37 °C. The media was aspirated from the top of the gels, 2.5 × 10^5^ modified β6-1089 cells suspended in 500 μl complete Ham’s F12 per gel were added on top of the gels and incubated for 4 hours at 37 °C. Then 2.5 × 10^5^ MDA-MB-231 or SUM159 cells in 500 μl complete DMEM per gel were added and incubated overnight at 37 °C.

Collagen-I-coated nylon membranes (100-μm pore size) were put on top of steel grids (coated side facing up) in a well of a 6-well plate and the organotypic gels placed on top. The well was filled with complete DMEM until the liquid reached the grid-membrane interface level; media was replenished every 2 days. The gels were harvested after 10 days, fixed in 10% neutral-buffered formalin (Cell Path, Newtown, UK, BAF-0010-037) overnight and then transferred to 70% ethanol for 24 hours. Gels were mounted in paraffin and sectioned.

### Invadopodia assay (in situ zymography)

To analyse MEC-derived gelatinase activity an in situ zymography assay was carried out. To prepare the gelatin solution, 178.12 mg NaCl (Thermo Fisher Scientific, BB358-1), 94.57 mg NaBH_4_ (Sigma-Aldrich, 21,346-2) and 100 mg porcine skin type A gelatin (Sigma-Aldrich, G2500) were dissolved in 50 ml PBS for 1 hour at 37 °C (pH 9.3), following which 1.8 mg rhodamine was added to the solution and mixed for 2 hours to fluorescently label the gelatin. The gelatin solution was dialysed against PBS for 48 hours using Slide-A-Lyzer Dialysis Cassettes with 10 K MWCO (Pierce, Rockford, IL, USA, 66830), the PBS being replenished every 24 hours. After dialysis, the gelatin was collected and centrifuged at 1200 rpm for 10 minutes to remove insoluble particles, and then 1 g sucrose (Thermo Fisher Scientific, 8060153) was added and dissolved. The rhodamine-conjugated gelatin was spun at 1200prm for 10 minutes and then heated to 37 °C. Forty microlitres of solutions (drops) were aliquoted onto a 10-cm tissue culture plate. Thirteen-millimetre coverslips were placed on each drop and incubated for 20 minutes. All incubation steps were performed in the dark. In another dish, 1% gluteraldehyde solution in PBS (v/v) was aliquoted as 40 μl drops. After 20 minutes gelatin-covered coverslips were placed on the gluteraldehyde solution to create a dual layer, and incubated for 15 minutes. The coverslips were placed in a 24-well plate (dual-layered side facing up) and washed three times with 500 μl PBS, after which 500 μl 5 μg/ml NaBH_4_ was added and incubated for 20 minutes then washed as described. For sterilisation, coverslips were incubated with 500 μl 70% ethanol for 5 minutes, washed with PBS, then equilibrated and free aldehydes quenched using 500 μl complete Ham’s F12 for 20 minutes at 37 °C. Finally, 4 × 10^4^ modified β6-1089 or N-1089 cells were added onto the coverslips and incubated for 24 hours.

After incubation, the cells were washed three times with 500 μl PBS and fixed in 4% formaldehyde (PFA, Sigma-Aldrich, P6148) for 15 minutes at room temperature then washed again as described. Cells were blocked in 0.1% BSA (v/v) and 0.1% NaN_3_ (Thermo Fisher Scientific, S227I) in DMEM (w/v) for 15 minutes in the dark at 4 °C, then stained with FITC-labelled phalloidin (Invitrogen, A22283) for 20 minutes in the dark at room temperature. The coverslips were mounted in ProLong Gold Antifade aqueous mounting reagent (Invitrogen, P36931) with DAPI.

### Immunoprecipitation of conditioned media

Two hundred microlitres of 20× concentrated CM samples was cleared with 20 μl Protein A sepharose beads (GE Healthcare, CL-4B) for 1 hour at 4 °C with rotation. The samples were spun at 6000 rpm for 2 minutes at 4 °C and supernatant were collected. Pre-cleared CM were divided into 100 μl aliquots and incubated with 5 mg MMP-8 antibody (R&D Systems, mAb 908) or mouse IgG (Sigma-Aldrich, I5381) overnight at 4 °C with rotation.

Antibody treated CM was added to fresh 20 μl beads and incubated for 4 hours at 4 ° C with rotation. Samples were centrifuged as described. Supernatant were collected and kept as unbound fraction. The remaining pellet was washed with serum-free Ham’s F12 as described. In order to remove the precipitates from the beads, 40 μl reducing Laemmli buffer was added to 20 μl cleared bead pellet and boiled at 100 °C for 10 minutes then spun once again. Supernatants were collected and kept as bound fraction. Laemmli buffer was added to unbound fraction and boiled at 100 °C for 5 minutes. Twenty microlitres of prepared sample of bound or unbound fractions were loaded and separated on an SDS acrylamide gel and transferred to nitrocellulose as previously described, and probed with anti-MMP-8 or anti-V5 antibody.

### Immunofluorescence staining

Cells on coverslips were fixed with 4% PFA in PBS for 10 minutes at room temperature (RT). Cells were washed three times with 500 μl PBS then blocked and permeabilized with 2% BSA and 0.1% Triton X-100 in PBS for 15 minutes. Finally, cells were incubated with primary antibody prepared in blocking solution at 1/100 dilution for 1 hour at room temperature. Primary antibodies used were α6β4 integrin (Merck Millipore, Billerica, MA, USA, MAB1964) and plectin (Epitomics, Burlingame, CA, USA, 1399). Cells were incubated with secondary antibody conjugated with FITC or Cy3 diluted in PBS. (Invitrogen, anti-mouse 546, A11030, anti-rabbit 546, A11035, anti-mouse 488, A11029, anti-rabbit 488, A11008, phalloidin 546, A22283). The cells were mounted in ProLong Gold Antifade aqueous mounting reagent with DAPI. For organotypic cultures, Neso antibody was a kind gift from Prof M. Djamgoz, Imperial College London.

For paraffin-embedded organotypic sections: sections were dewaxed and rehydrated, then boiled in 10 mM Tri-sodium citrate (pH 6) for 20 minutes. Sections were then incubated in 0.5% Triton X-100 for 5 minutes at RT. After that the sections were washed with PBS for 1 minute (three times) and blocked with 5% BSA/PBS for 1 hour at room temperature. Primary antibodies (p63; Dako, M7247, 1/50, Neso; 1/300, Ki67; Novocastra, Newcastle upon Tyne, UK, NCL-L-Ki67-MM1, 1/100) were prepared in blocking solution and incubated for 1 hour at room temperature. After that sections were washed with PBS for 5 minutes (three times). Fluorescently tagged secondary antibodies were prepared in PBS and incubated for 45 minutes at RT. Finally, the sections were washed as described followed by an extra washing step with distilled water and mounted in ProLong Gold Antifade reagent with DAPI. The Ki67 index is calculated as the percentage of Ki67-positive invading breast cancer cells out of the total cell number.

### Luciferase reporter assay

MDA-MB-231 cells transfected with firefly and Renilla luciferase reporter gene fused with PAI-promoter (MDA-MB-231-Luc) were a gift from Dr Caroline Hill (London Research Institute) and cultured in DMEM in the presence of 50 μg/ml blasticidine (Sigma-Aldrich, 15205). MDA-MB-231- Luc cells were seeded onto 96-well plates at a density of 4 × 10^4^ cells per well and incubated overnight at 37 °C, followed by serum starvation for 4 hours. 5 × 10^4^ modified β6-1089 or N-1089 cells were seeded on top of MDA-MB-231-Luc cells in SFM and co-cultured overnight. The media was removed and cells were washed with 100 μl PBS. Cell lysis and luciferase activity quantification was performed using the Dual-Luciferase Reporter Assay System (Promega, E-1910) according to the manufacturer’s instructions.

### Zymography

CM collected from modified β6-1089 or N-1089 cells was concentrated as previously described and separated by SDS-PAGE gel containing 3 mg Collagen-I for 50 minutes at 200 V. The gel was developed with developing buffer [10 ml 1 M Tris (pH 7.5), 8 ml 5 M NaCl (Thermo Fisher Scientific, BP358), 1 ml 1 M CaCl_2_ (Sigma-Aldrich, C7902), 1.6 ml 2.5%Triton X-100 and 179.4 ml sterile distilled water] overnight at 37 °C and stained with Coomassie Blue R-250 solution (Thermo Fisher Scientific, 20278).

### Statistical analysis

Statistical significance was determined by Student’s *t* test or ANOVA with Bonferroni post-test where appropriate, using Prism (Graphpad Software, San Diego, CA, USA). For immunohistochemical scoring of MMP8 on a duct-by-duct basis Fisher’s exact test was used on a 2 × 3 table. Results were considered as significant with *P* value less than 0.05.

## Results

### MMP-8 is expressed by normal myoepithelial cells and is lost in DCIS-associated myoepithelial cells

**Fig. 1:**
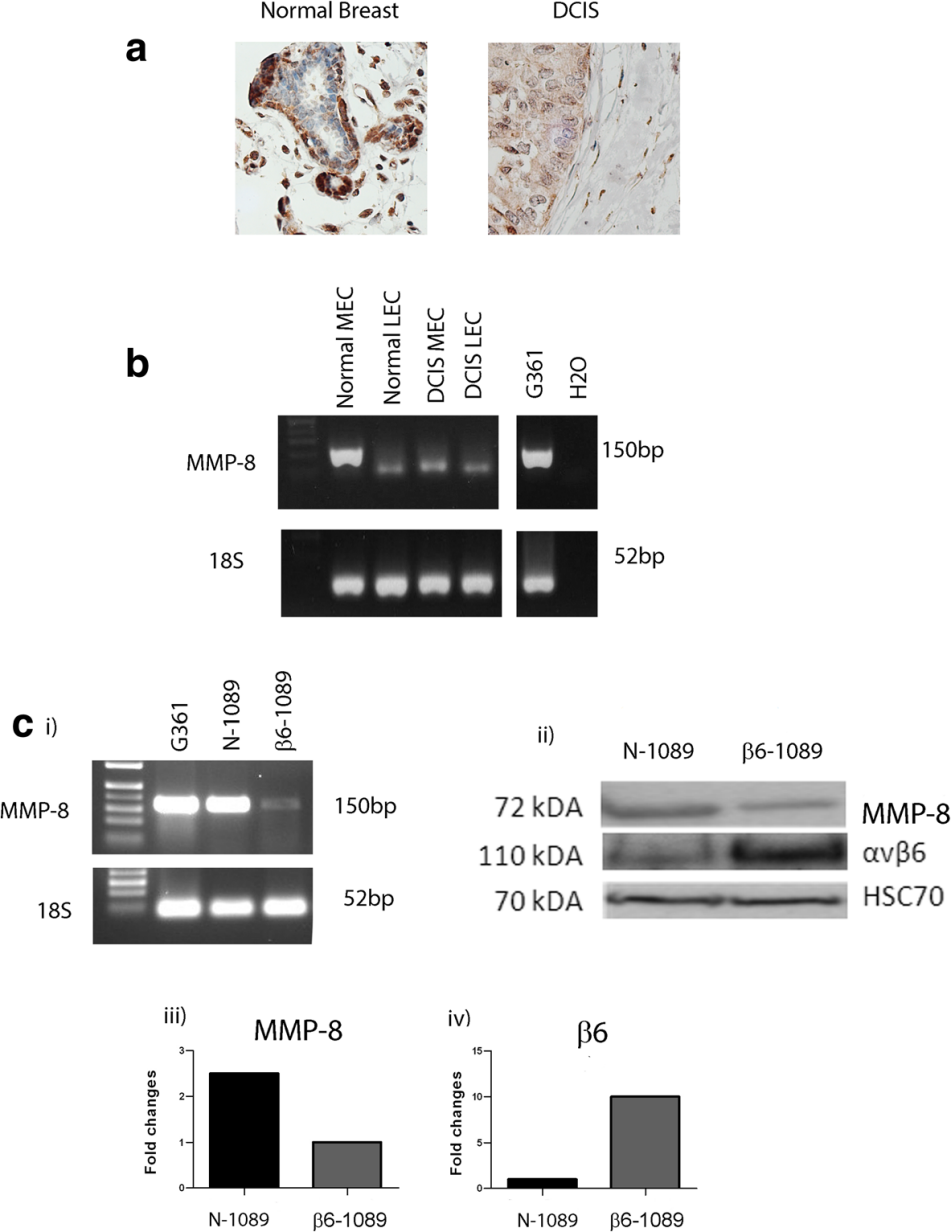
MMP-8 expression in; primary normal and DCIS myoepithelial cells and a cell line model. **a** Normal and ductal carcinoma in situ (*DCIS*) tissue immunohistochemically stained for matrix metalloproteinase-8 (*MMP-8*) (Atlas, HPA02122, 1:1000). Clear MMP-8 staining can be observed in the normal myoepithelial cells (*MECs*), which is absent in the DCIS section. Magnification × 400. **b** Normal MECs and luminal epithelial cells (*LECs*) were isolated from reduction mammoplasty tissue and DCIS MEC and DCIS LEC were isolated from a patient with high-grade DCIS. Tissues were enzymatically digested and magnetic bead separation was used to isolated specific populations (MEC - ITGB4, LEC - EpCAM). RNA was extracted from the isolated cells and G361 cells (a positive control for MMP-8), converted to cDNA by reverse transcription and then subjected to nested PCR for MMP-8 or PCR of housekeeping gene 18S. The MMP-8 PCR produces a product of 150 bp in normal MEC and G361 lanes, and other lanes only give a weak band. 18S gives a size of 52 bp in all sample lanes. The water (H_2_O) control lane shows no band. **c** (i) RNA was extracted from cell lines; G361, N-1089 and β6-1089 and converted to cDNA as above. The cDNA was subjected to nested PCR for MMP-8 and bands were identified in G361 and N-1089 but only a weak band was observed in the β6-1089 lane. All samples demonstrated a band for 18S. (ii) RIPA protein lysates were produced from N-1089 and β6-1089 cells and 20 ug of protein was run on 8% PAGE then transferred to nitrocellulose and probed for MMP-8 (R&D Systems, mAb 908) αvβ6 (Santa Cruz Biotechnology, SC-6632) or HSC70 as a loading control. The gel indicates that N-1089 expresses higher levels of MMP-8 and much lower levels of αvβ6. While β6-1089 exhibit much higher levels of αvβ6 and lower levels of MMP-8. (iii) Densitometry of western blots indicates that MMP-8 expression in N-1089 is 2.5-fold higher than N-1089 and (iv) αvβ6 expression in β6-1089 is 10-fold higher than N-1089

In order to confirm previous observations that the primary source of MMP-8 in normal breast is the MEC population and that it is lost in DCIS-associated MECs, we first undertook to stain normal and DCIS tissue for MMP-8. It can be seen in representative images in Fig. 1a that normal MECs express MMP-8 while MECs associated with DCIS do not. Ducts from seven reduction mammoplasty samples were homogenously positive for MMP-8 (as was hyperplasia) (Additional file 1: Table S1). Thirty-one of 68 (45%) ducts from nine cases of pure DCIS (comprising high, intermediate and low grade) were negative for MMP-8. Thirty-nine of 48 (81%) of ducts from nine cases of DCIS with invasion were negative for MMP-8 (Additional file 2: Table S2). These data indicate a progressive loss of myoepithelial expression of MMP-8 from normal breast tissue to pure DCIS to DCIS with invasion.

Subsequently, primary normal MECs, luminal epithelial cells (LECs), fibroblasts and their DCIS-associated counterparts were isolated from normal breast and pure DCIS tissue respectively, as described [38], and a nested PCR for MMP-8 was carried out. Normal breast MECs exhibit expression of MMP-8 whereas this expression is lost in DCIS-associated MECs. MMP-8 expression was not detected in LECs derived from either normal or DCIS tissue (Fig. 1b) or fibroblast populations (not shown). In addition immunofluorescent dual labelling of the myoepithelial-specific protein p63 and MMP-8 demonstrates predominant myoepithelial localisation of MMP8 in a normal breast duct (Additional file 3: Figure S1).

One of the most consistent changes in DCIS-associated MECs is upregulation of the integrin αvβ6 [41]. Therefore, to establish a model for DCIS-associated MECs, a MEC line over-expressing αvβ6 integrin (β6-1089) was generated from a normal, αvβ6-negative MEC line (N-1089), as described [27]. To confirm that the β6-1089 line recapitulates the in vivo phenotype of DCIS, RT Q-PCR for MMP-8 was undertaken in both β6-1089 and N-1089 cell lines. MMP-8 expression was downregulated in β6-1089 cells as compared to N-1089 cells at both the mRNA (Fig. 1c i and iii) and protein level (Fig. 1c ii and iv), thus reflecting the changes seen in human tissue.

### MMP-8 alters adhesion and migration in DCIS-modified myoepithelial cells in a proteolytic-dependent manner

**Fig. 2:**
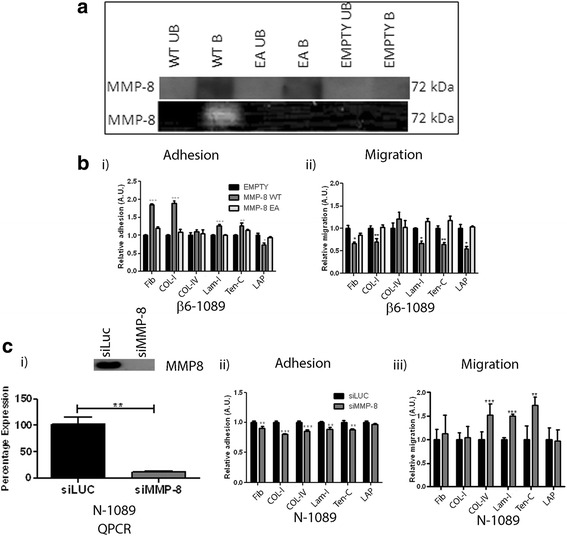
MMP-8 expression alters myoepithelial cell adhesion and migration to ECM proteins. **a** *Upper panel*: immunoprecipitation of matrix metalloproteinase-8 (*MMP-8*) from 20× concentrated conditioned media of β6-1089 transiently transfected with wild-type MMP-8 (*WT*), inactive point mutant of MMP-8 (*EA*) or empty vector (*EMPTY*). Conditioned media (CM) was incubated overnight with MMP-8 antibody (R& D Systems, mAb 908) or IgG (Sigma-Aldrich) and bound proteins were precipitated with Protein A beads (*B*) and unbound supernatant was also collected (*UB*). Samples were separated by 10% SDS-PAGE and probed with anti-V5 antibody directed to the V5 tag on the transfected MMP-8. *Lower panel*: Collagen-I zymography. CM was collected from the β6-1089 cells as described above and concentrated 20× using 3 K spin columns. Equal volumes were run by SDS-PAGE containing 3 mg of Collagen-I. The gel was incubated overnight in substrate solution and then stained with Coomassie. Only the lane containing CM from β6-1089 transfected with WT MMP-8 demonstrated a clear band indicating Collagen degradation at the same molecular weight as MMP-8. **b** (i) Adhesion to ECM proteins. β6-1089 cells transiently transfected with empty vector (EMPTY), wild-type MMP-8 (WT) or inactive mutant MMP-8 (EA) were plated on Fibronectin (*Fib*), Collagen-I (*Col-I*), Collagen-IV (*Col-IV*), Laminin-I (*Lam-I*), Tenascin-C (*Ten-C*), latency-associated peptide (*LAP*) or BSA (Control) for 1 hour. Then cells were treated with Calcein AM for 15 minutes before reading the fluorescence. The adhesion is calculated relative to the EMPTY. Expression of WT MMP-8 increased cell adhesion to Fib, Col-I, Lam-I and Ten-C, and decreased adhesion to LAP. The EA transfected cells did not show alterations in adhesion.(ii) β6-1089 cells treated in the same way as above were plated onto transwells coated on the underside with the same ECM proteins and cells were allowed to migrate for 8 hours before quantifying the number of cells that were on the top and bottom of the transwell. Migration was calculated relative to the EMPTY. Expression of WT MMP-8 decreased migration towards Fib, Col-I, Lam-I, Ten-C and LAP, while expression of the EA had no effect. **c** (i) N-1089 cells were transfected with siRNA targeting MMP-8 (siMMP-8) or Luciferase (siLuc) as a control. Ninety-six hours after transfection protein and RNA were collected and analysed by western blot and RT Q-PCR respectively. Both the western blot (*upper panel*) and RT Q-PCR (*graph*) indicate a reduction in MMP-8 at the protein and mRNA level in response to siMMP-8 treatment as compared to the siLUC control. (ii) N-1089 cells treated with siMMP-8 as described were plated onto the same selection of ECM proteins as previously described in (**b**). A significant reduction in adhesion to Fib, Col-I, Col-IV, Lam-I and Ten-C was observed in N-1089 transfected with siMMP-8 as compared to N-1089 transfected with siLUC. (iii) N-1089 cells treated with siMMP-8 were also plated on transwells coated with the same selection of ECM proteins as described in (**b**). The N-1089 transfected with siMMP-8 demonstrated a significant increase in migration towards Col-IV, Lam-I and Ten-C as compared to N-1089 + siLUC. ^**^ *p* = 0.01, ^***^ *p* = 0.001 (Student’s *t* test). Error bars = SEM, *n* = 3 independent experiments

To analyse the gain-of-function effect of MMP-8 in our model of DCIS-modified MEC, MMP-8 was re-introduced into β6-1089 cells by plasmid over-expression (MMP-8 WT). To dissect the involvement of proteolytic activity of MMP-8 in its biological role, cells were transfected with an enzymatically inactive form of MMP-8 (MMP-8 EA), generated by a point mutation in the catalytic domain in which an essential glutamic acid residue is changed to alanine [39]. In order to compare protein expression levels, conditioned media (CM) were collected from cells transfected with MMP-8 WT, MMP-8 EA or empty pcDNA4 vector, then concentrated 20×, and immunoprecipitated for MMP-8. Unbound and immunoprecipitated (bound) fractions were subjected to western blotting and similar protein expression levels for WT and EA were observed, with no detection in vector-only CM (Fig. 2a *upper panel*). MMP-8 is a major collagenase that cleaves Collagen-I [21] therefore the degradative activity of WT and EA forms was examined using Collagen-I zymography. Degradation of Collagen-I was observed only by CM from MMP-8 WT, while MMP-8 EA or empty vector control failed to show any degradation (Fig. 2a *lower panel*). This confirms the inability of the EA form to cleave Collagen-I.

To analyse whether MMP-8 is involved in MEC adhesion to ECM; MMP-8 WT, MMP-8 EA or control cells were seeded onto different ECM and tracked fluorescently by Calcein AM cell tracker. After 1 hour, non-adherent cells were washed and fluorescence generated from adherent MMP-8 WT and MMP-8 EA cells was normalised to empty vector. Over-expression of MMP-8 WT significantly enhanced adhesion to Fibronectin (Fib), Collagen-I (Col-I), Laminin-I (Lam-I or Lam αI-βI-γI) and Tenascin-C (Ten-C) whereas there was a significant decrease in adhesion to the N terminus latency-associated peptide of transforming growth factor beta (TGF-β) (LAP) (Fig. 2b i). LAP is an established ligand for αvβ6 integrin whereby LAP cleavage by αvβ6 integrin leads to TGF-β activation [42]. In contrast, MMP-8 EA cells, which lack proteolytic activity, showed no evidence of increased ECM adhesion compared to control cells.

To explore the effect of MMP-8 over-expression on MEC migration towards ECM, transwell migration assays were performed. In this system MECs are separated from ECM via a cell-permeable membrane and cells move towards the ECM (Fig. 2b ii). Migration towards Fib, Col-I, Lam-I, Ten-C and LAP was significantly reduced in MMP-8 WT but not in MMP-8 EA cells compared to control empty vector transfected cells. The inability of MMP-8 EA to exert an effect on adhesion or migration indicates that enzymatic activity of MMP-8 is necessary for its function.

To study the loss-of-function effect of endogenous MMP-8 in our normal N-1089 cells, MMP-8 expression was knocked down by siRNA transfection. Knock-down of MMP-8 was confirmed at mRNA and protein level (Fig. 2c i). Adhesion of MEC to Fib, Col-I, Col-IV, Lam-I and Ten-C was significantly reduced in the absence of MMP-8 (Fig. 2c ii) though interestingly there was no significant difference in adhesion to the αvβ6 ligand LAP.

Transwell migration assays were used to analyse the effect of lack of endogenous MMP-8 on directed migration. MMP-8 knock-down in N-1089 cells led to increased migration towards Col-IV, Lam-I and Ten-C while there was no significant alteration in migration towards other ECM molecules (Fig. 2c iii).

### MMP-8 WT modulates α6β4 integrin localisation

**Fig. 3:**
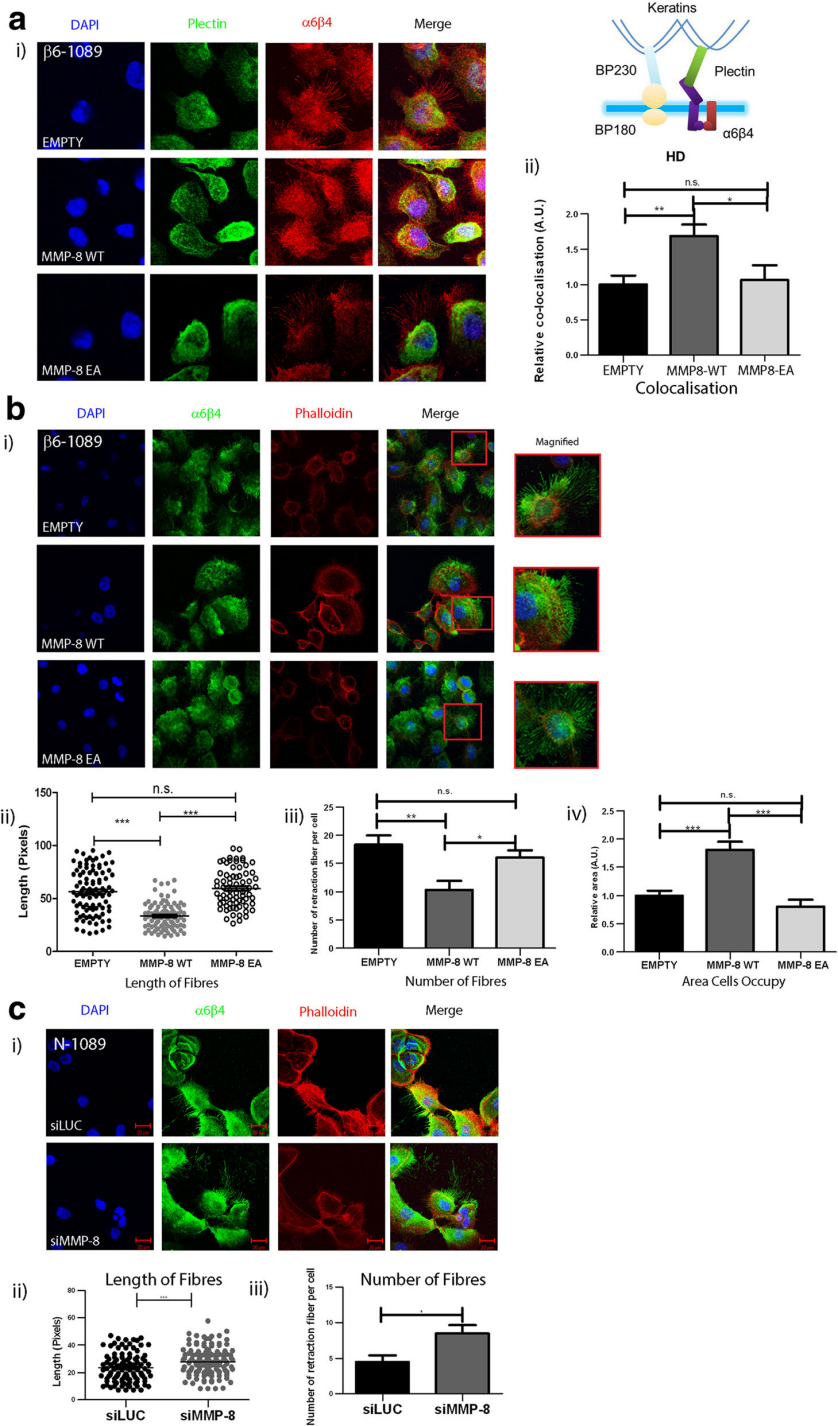
MMP-8 expression alters α6β4 subcellular distribution and myoepithelial cell morphology. **a** (i) Confocal images of β6-1089 cells transfected with empty vector (*EMPTY*) wild-type matrix metalloproteinase-8 (*MMP-8 WT*) or inactive mutant MMP-8 (*EA*) and grown on Fibronectin for 24 hours then fixed and co-stained for α6β4 (*red*) and plectin (*green*). Insert: cartoon illustrating hemidesmosome (*HD*) components, indicating the integrin α6β4 associates with plectin to link the complex to the keratin cytoskeleton. (ii) Co-localisation of α6β4 and plectin was determined by quantifying the number of yellow pixels per field of view in each condition then expressed as a ratio as compared to EMPTY. β6-1089 transfected with WT demonstrated a significant increase in α6β4 and plectin co-localisation, while the β6-1089 transfected with EA exhibited no change as compared to EMPTY. **b** (i) Confocal images of β6-1089 cells transfected with empty vector (EMPTY) wild-type MMP-8 (WT) or inactive mutant MMP-8 (EA) and grown on Fibronectin for 24 hours then fixed and co-stained for α6β4 (*green*) and phallodin (*red*). (ii) Morphological features of the transfected β6-1089 were analysed and length of trailing fibres was noted to be altered. Analysis of the length of these fibres indicated that β6-1089 transfected with MMP-8 WT significantly reduced the fibre length as compared to β6-1089 transfected with empty vector or MMP-8 EA. (iii) Analysis of the number of these fibres per cell also demonstrated a significant reduction in β6-1089 transfected with MMP-8 WT as compared to β6-1089 transfected with empty vector or MMP-8 EA. (iv) The final morphological alteration analysed was the physical space the cells occupied, the relative area occupied by β6-1089 transfected with MMP-8 WT was significantly larger than that occupied by β6-1089 transfected with empty vector or MMP-8 EA. **c** (i) Confocal images of N-1089 cells transfected with siRNA targeting MMP-8 (*siMMP-8*) or Luciferase (*siLuc*) as a control grown on Fibronectin for 24 hours then fixed and co-stained for α6β4 (*green*) and phallodin (*red*). (ii) Trailing fibre length was significantly longer in N-1089 transfected with siMMP8 as compared to siLUC. (iii) There were significantly more trailing fibres per cell in the N-1089 + siMMP8 group as compared to N-1089 transfected with siLUC. ^*^ *p* = 0.05 ^**^ *p* = 0.01, ^***^ *p* = 0.001 (Student’s *t* test). Error bars = SEM, *n* = 3 independent experiments

To further investigate the mechanism by which MMP-8 might influence MEC adhesion and migration, the expression and distribution of a major MEC integrin, α6β4 was examined by immunofluorescence. α6β4 integrin is a key component of HDs, which are the adhesive structures responsible for stable attachment of the basal cell surface of MEC to the basement membrane (Fig. 3a insert). One unique alteration reported in DCIS MECs is the disassociation of these structures [43, 44], α6β4 localising to invasion-associated actin complexes rather than the adhesive plectin complex [45]. Confocal analysis of dual-labelling for α6β4 and the HD component plectin revealed that α6β4 localises more to HD structures in MMP-8 WT compared to MMP-8 EA or control cells (Fig. 3a i). Dual staining of α6β4 with phalloidin showed that α6β4 also localises to actin-rich protrusion sites (Fig. 3b i) and these retraction fibres were significantly shorter and reduced in number in MMP-8 WT compared to either EA or empty vector cells (Fig. 3b ii and iii) consistent with the less migratory phenotype seen in MMP-8 WT MECs. In addition, on Fibronectin matrix, MMP-8 WT cells showed a greater degree of spreading with a significant increase in occupied area (in pixels) compared to MMP-8 EA or empty vector control cells (Fig. 3b iv).

Immunofluorescence analysis of N-1089 cells following MMP-8 knock-down revealed an increase in the length and number of retraction fibres compared to control knock-down cells, suggesting that MMP-8 inhibits the formation of these fibres (Fig. 3c i, ii and iii).

### Myoepithelial MMP-8 WT but not MMP-8 EA reduces breast cancer cell invasion in 2D system via paracrine interactions

**Fig. 4:**
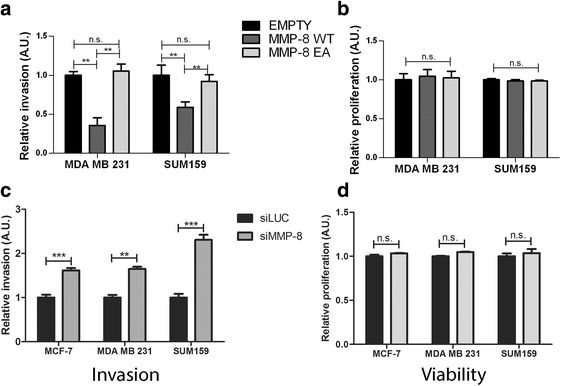
MMP-8 expression by myoepithelial cells has a paracrine effect on tumour cell invasion. **a** β6-1089 cells transfected with empty vector (*EMPTY*) wild-type matrix metalloproteinase-8 (*MMP-8 WT*) or inactive mutant MMP-8 (*EA*) were plated into 24-well plates and grown in serum-free media for 24 hours. Transwells coated with Matrigel were placed into the wells and MDA-MB-231 or SUM159 cells were placed on top of the Matrigel. After 24 hours the transwells were removed and the number of invading cancer cells counted. Invasion was calculated relative to the β6-1089 transfected with empty vector. MDA-MB-231 and SUM159 cells exhibited a significant reduction in invasion when in the presence of β6-1089 transfected with WT cells, this was not observed with the β6-1089 transfected with EA cells. **b** MTS assay for viability on MDA-MB-231 and SUM159 cells grown for 24 hours in the presence of conditioned media (CM) from β6-1089 cells transfected with empty vector (EMPTY) wild-type MMP-8 (WT) or inactive mutant MMP-8 (EA). No difference was observed between any of the groups relative to β6-1089 transfected with empty vector. **c** Transwells were set up as described above with N-1089 cells transfected with siRNA targeting MMP-8 (*siMMP-8*) or Luciferase (*siLuc*) were plated into the bottom wells. MCF-7, MDA-MB-231 and SUM159 cells were plated into Matrigel-coated transwells above. After 24 hours (or 48 hours for MCF-7) the transwells were removed and the number of invading cancer cells counted. Invasion was calculated relative to the N-1089 transfected with siLUC. MDA-MB-231 and SUM159 cells exhibited a significant increase in invasion when in the presence of N-1089 transfected with siMMP-8. **d** MTS assay for viability on MCF-7, MDA-MB-231 and SUM159 cells grown for 24 hours in the presence of conditioned media (CM) from N-1089 cells transfected with siRNA targeting MMP-8 (siMMP-8) or Luciferase (siLuc). No difference was observed between the two groups. ^**^ *p* = 0.01, (Student’s *t* test). Error bars = SEM, *n* = 3 independent experiments

Normal breast MECs previously have been shown to reduce breast cancer cell invasion through paracrine mechanisms [13]. Transwell invasion assays were used to assess the involvement of MEC-derived MMP-8 in the cross-talk between MECs and breast cancer cells during early invasion through basement membrane, MDA-MB-231 or SUM159 breast cancer cells were seeded onto transwells coated with Matrigel and co-cultured with β6-1089 cells expressing MMP-8 WT, MMP-8 EA or empty vector control plated onto outer chamber for 24 hours (Fig. 4). A significant reduction in the number of breast cancer cells invading through Matrigel in the presence of β6-1089 cells expressing MMP-8 WT was observed compared to cells transfected with EA or empty vector (Fig. 4a). Transwells carried out using ER+ cell line MCF-7 showed no reduction in invasion (data not shown) – however these cells exhibited a low degree of invasion and therefore it can be speculated that the invasion could not be reduced any further. These results indicate that the invasion-suppressor activity exhibited by MMP-8 WT MECs is dependent upon MMP-8 enzymatic activity, as MMP-8 EA does not influence tumour cell invasion. To show a direct effect of MMP-8 on breast cancer cell invasion, recombinant MMP-8 containing serum-free media was used in the outer chamber, which resulted in a significant decrease in breast cancer cell invasion similar to that seen with MMP-8 WT MECs (data not shown). In order to eliminate the possibility that the reduced invasion was a reflection of altered cell number of breast cancer cells, MDA-MB-231 and SUM159 cells were treated with CM collected from modified MECs after 24 hours of starvation (the same time duration as the invasion assays). The tumour cells were analysed in an MTS assay, which revealed no differences in relative cell number of the MDA-MB-231 or SUM159 cells (Fig. 4b). When the MMP-8 substrate Col-I was replaced with Matrigel in transwell invasion assays, there also was a significant downregulation in breast cancer cell invasion towards MMP-8 WT over-expressing β6-1089 cells, but not MMP-8 EA or empty vector transfected cells (data not shown).

To dissect whether loss of normal MEC-derived MMP-8 has an influence on breast cancer cell invasion, MCF-7, MDA-MB-231 and SUM159 cells were co-cultured with N-1089 control cells following knock-down of MMP-8 in a transwell setup. This demonstrated that breast cancer cell invasion was significantly enhanced in the absence of MMP-8 (Fig. 4c). These data support the earlier contention that reduction in MCF-7 invasion was too small to measure, as co-culturing with MECs knocked down for MMP-8 enhanced MCF-7 invasion significantly. No significant difference in breast cancer cell number was demonstrated in the presence of N-1089 control or MMP-8 knock-down CM (Fig. 4d), supporting the concept that MMP-8 suppresses invasion and does not alter cell number.

### Myoepithelial MMP-8 WT suppresses breast cancer cell invasion in 3D organotypic culture

**Fig. 5:**
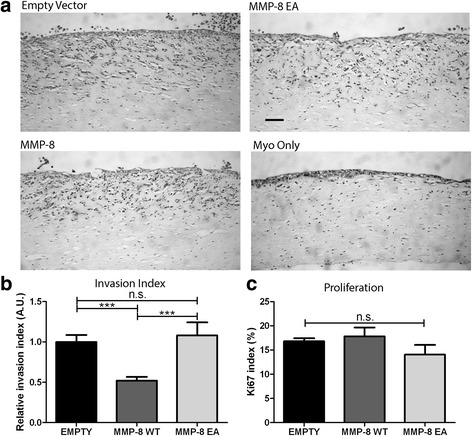
MMP-8 expression in myoepithelial cells alters tumour cell invasion in 3D organotypics. **a** 3D organotypic microenvironment of the breast was created by mixing collagen and Matrigel with normal breast fibroblasts. β6-1089 cells transfected with empty vector (*EMPTY*) wild-type matrix metalloproteinase-8 (*MMP-8 WT*) or inactive mutant MMP-8 (*EA*) were plated on top of the gel and allowed to attach, then MDA-MB-231 cells were plated on top of the transfected β6-1089 cells. Organotypics were grown at an air/liquid interface for 10 days then fixed and embedded in paraffin. Section were taken and stained with H&E and images were taken for analysis. **b** An invasion index was determined for each condition by multiplying; the average depth of invasion (from multiple points in each section), the number of invading aggregates and the size of the aggregates. MDA-MB-231 cells grown with β6-1089 transfected with WT exhibited a significantly reduced invasion index relative to the β6-1089 transfected with EMPTY and EA. **c** Organotypic sections were stained for Ki67 and the positively staining cells were quantified to determine the level of proliferation in each condition. There was no difference observed between any of the groups. ^***^ *p* = 0.001 (Student’s *t* test). Error bars = SEM, *n* = 3 independent experiments

In order to recapitulate the DCIS-related stromal microenvironment, 3D organotypic cultures were constructed from Col-I/Matrigel gels embedded with primary normal breast fibroblasts and overlaid with modified MECs and MDA-MB-231 or SUM159 breast cancer cells, to reflect the juxtapositioning of these cell populations in breast tissue (Fig. 5). After 10 days of incubation, gels were fixed, sectioned and stained with hematoxylin and eosin (H&E) (Fig. 5a). In order to discriminate the invading breast cancer cells from modified β6-1089 cells, organotypic cultures were stained for the invasive breast cancer cell marker Neso [46], which selectively detects the tumour cells invading into the gel (Additional file 4: Figure S2). An invasion index was quantified by combining the depth, number and area occupied by invaded cells and normalised to invasion observed when breast cancer cells were co-cultured with β6-1089 cells transfected with empty vector control. There was a significant decrease in tumour cell invasion only in the presence of MMP-8 WT transfected β6-1089 cells (Fig. 5b). To confirm that the reduced invasion index does not reflect decreased proliferation, organotypic gels were stained for the proliferation marker Ki67. The Ki67 index indicates that proliferation is not significantly altered in any of the transfection groups (Fig. 5c).

### MMP-8 modifies MMP-9 activity

**Fig. 6:**
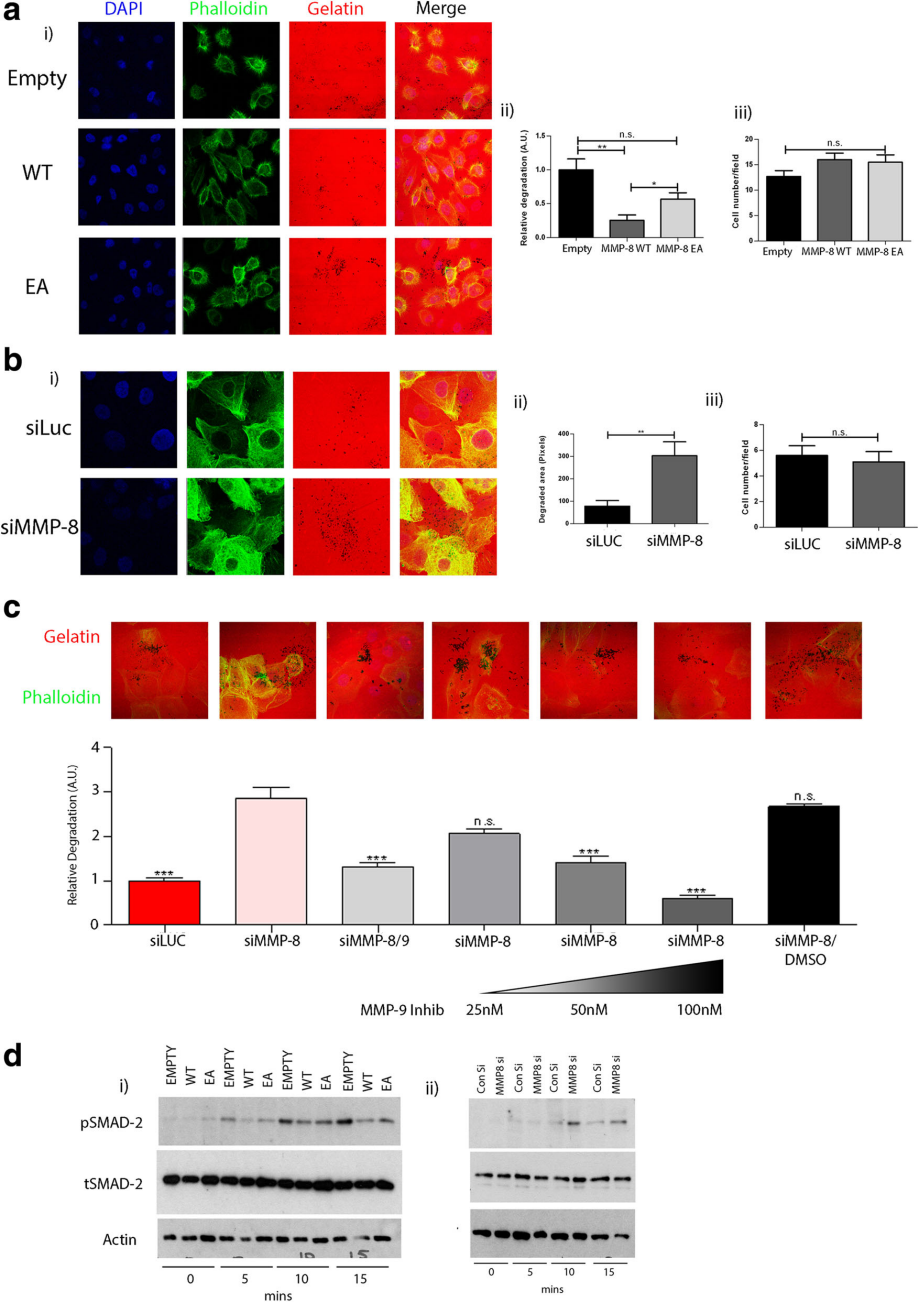
MMP-8 expression in myoepithelial cells alters MMP-9 expression/function and TGF-β signalling. **a** (i) Confocal images of β6-1089 cells transfected with empty vector (*EMPTY*) wild-type matrix metalloproteinase-8 (*MMP-8 WT*) or inactive mutant MMP-8 (*EA*) and grown on rhodamine-tagged gelatin (*red*) for 24 hours then fixed and stained with FITC-conjugated phallodin (*green*). (ii) Gelatinase activity was quantified by measuring the total size of the black spots (degraded gelatin) per field of view relative to the β6-1089 transfected with EMPTY. There was a significant reduction in gelatin degradation in β6-1089 transfected with WT and EA compared to β6-1089 transfected with EMPTY, albeit the reduction in β6-1089 transfected with EA was not as marked. (iii) The number of cells per field of view was determined and no significant difference was noted between the groups. **b** (i) Confocal images of N-1089 cells transfected with siRNA targeting MMP-8 (*siMMP-8*) or Luciferase (*siLuc*) and grown on rhodamine-tagged gelatin (*red*) for 24 hours then fixed and stained with FITC-conjugated phallodin (*green*). (ii) Gelatin degradation was significantly increased in N-1089 transfected with siMMP-8 as compared to siLUC. (iii) Quantification of the number of cells per field of view indicated no difference between the two groups. **c** Confocal images of N-1089 cells transfected with siRNA targeting MMP-8 (siMMP-8) and MMP-8 and MMP-9 (siMMP-8/9) or Luciferase (siLuc) and grown on rhodamine-tagged gelatin (*red*) for 24 hours with or without an MMP-9 inhibitor at increasing concentration then fixed and stained with FITC-conjugated phallodin (*green*). The graph indicates relative gelatin degradation increases in N-1089 transfected with siMMP-8, this increase is significantly reduced when both MMP-8 and MMP-9 are knocked down (siMMP-8/9). The increase in gelatin degradation can also be significantly reduced, in a dose-dependent manner, with an MMP-9 inhibitor. **d** (i) Western blots for SMAD-2 on protein extracted from β6-1089 cells transfected with empty vector (EMPTY), wild-type MMP-8 (WT) or inactive mutant MMP-8 (EA) after treatment with TGF-β (5 ng/ml) for 5, 10 and 15 minutes. β6-1089 transfected with empty vector exhibit higher levels of Phospho (p)SMAD-2 at all time points than the β6-1089 transfected with WT, while the β6-1089 transfected with EA appear to show moderate expression. Total (t)SMAD-2 levels are similar in all lanes as is the actin loading control. (ii) Western blots for SMAD-2 on protein extracted from of N-1089 cells transfected with siRNA targeting MMP-8 (siMMP-8) or Luciferase (siLuc) after treatment with TGF-β (5 ng/ml) for 5, 10 and 15 minutes. N-1089 transfected with siMMP-8 exhibit higher expression of pSMAD-2 at 10 and 15 minutes than the N-1089 transfected with siLUC. The tSMAD2 and actin expression levels appear to be consistent. ^*^ *p* = 0.05 ^**^ *p* = 0.01, ^***^ *p* = 0.001 (Student’s *t* test). Error bars = SEM, *n* = 3 independent experiments

We previously have identified increased MMP-9 activity in β6-1089 cells compared to N-1089, downstream of enhanced TGF-β signalling [41]. Furthermore it has been reported that MMP-9 can cleave the extracellular domain of α6β4 and in turn interfere with HD assembly [47]; we therefore analysed the expression levels and proteolytic function of MMP-9 in β6-1089 cells, with and without MMP-8 over-expression. RT Q-PCR revealed no significant difference in MMP-9 mRNA levels between empty, MMP-8 WT or MMP-EA transfected cells (data not shown). To study whether the proteolytic activity of MMP-9 is altered upon MMP-8 expression, modified β6-1089 cells were grown on rhodamine-labelled MMP-9 substrate gelatin for 24 hours and then fixed and stained with FITC-conjugated phalloidin. The degraded matrix area was quantified, and demonstrated a significant reduction in gelatin degradation in β6-1089 cells transfected with MMP-8 WT compared to the EA form or empty vector (Fig. 6a i and ii). There was no difference in cell number per field among different transfection groups (Fig. 6a iii).

In order to understand whether MMP-8 loss in N-1089 cells can influence MMP-9 function, modified N-1089 cells were seeded on MMP-9 substrate as described and gelatinase activity was quantified. The area of degraded gelatin was significantly increased in MMP-8 knocked down cells, with no change in cell number (Fig. 6b i-iii).

To further investigate the MMP-9 activity, MMP-9 was knocked down by siRNA treatment in the MMP-8 knocked down N-1089 cells (MMP-9 knock-down was verified by RT Q-PCR, data not shown) or cells were treated with an MMP-9 inhibitor (Fig. 6c). Gelatin degradation was significantly downregulated upon MMP-9 knock-down in siMMP-8-treated N-1089 cells. In addition, there was a dose-dependent decrease in degraded gelatin area in relation to increasing concentrations of MMP-9 inhibitor. There was no difference in gelatin degradation in DMSO vehicle control compared to siMMP-8 treatment suggesting that the increased gelatin degradation is derived from increased MMP-9 activity after MMP-8 knock-down.

### MMP-8 reduces TGF-β signalling

To study the effect of MMP-8 expression on TGF-β ignalling, modified β6-1089 cells were treated with 5 ng/ml active TGF-β1 for 5, 10 and 15 minutes. As a readout for TGF-β signalling activity, the phosphorylation levels of TGF-β downstream element SMAD-2 was evaluated by WB. The expression levels of total and phosphorylated forms of SMAD-2 were normalised to that of actin. The phosphorylation levels of SMAD-2 were decreased in β6-1089 cells over-expressing MMP-8 WT as compared to empty vector control and EA mutant cells at 5 minutes. There also was a reduction in phospho-SMAD-2 in β6-1089 cells over-expressing MMP-8 EA as compared to empty vector at 15 minutes. The phosphorylation levels of SMAD-2 in MMP-8 EA were higher than phospho-SMAD-2 levels in MMP-8 WT at 5 minutes (Fig. 6d i and Additional file 5: Figure S3i).

To investigate the effect of loss of MMP-8 on TGF-β downstream signalling, modified N-1089 cells, with MMP-8 knock-down, were treated with active TGF-β1 as described. This revealed an increase in phosphorylated SMAD-2 in MMP-8 knocked-down N-1089 cells compared to control siRNA transfected cells at 5, 10 and 15 minutes (Fig. 6d ii and Additional file 5: Figure S3ii), suggesting that MMP-8 reduces TGF-β signalling, and this may not be dependent on enzymatic activity.

## Discussion

Transition of DCIS to invasive cancer is a critical step in the natural history of breast cancer, converting a potentially curable lesion to a life-threatening disease. Only around half of DCIS lesions will progress to invasive cancer during a woman’s lifetime, but there is a lack of robust predictive markers to stratify patient management [1, 48, 49]. Molecular studies have indicated a consistent similarity between DCIS tumour cells and their invasive counterparts, showing that DCIS cells are as genetically advanced as invasive cancer. This has focused attention on the microenvironment, which shows evidence of being significantly altered in DCIS compared to normal tissue [6, 7, 50, 51]. A unique component of the breast microenvironment is the MEC population. In the normal breast, MECs form a stable interface with the basement membrane, regulate epithelial cell polarity and exert a multifaceted tumour-suppressor role [9, 52–55]. We, and others, have shown that MECs are altered in DCIS and we recently demonstrated that upregulation of the integrin αvβ6 by DCIS-associated MEC confers tumour-promoter activity on MECs through TGF-β-mediated upregulation of MMP-9 [27].

Whilst many MMPs exert tumour-promoter effects, a greater understanding of metalloproteinase biology has indicated that some family members have tumour-suppressor effects. MMP-8 was identified as a tumour suppressor nearly a decade ago when the MMP-8 knock-out mouse was found to be more susceptible to skin tumuorigenesis as compared to a WT mouse [28]. There is also strong evidence for a role in tumour prevention in breast cancer, melanoma and squamous cell carcinoma [22, 26, 28, 29, 31, 32, 56–59]. Particularly in melanoma; MMP-8 is frequently mutated by loss of heterozygosity (LOH), which is a hallmark for tumour-suppressor proteins [22]. In addition this mutation results in a significant decrease in enzymatic activity and a concomitant loss of its cancer-protective role [26]. However understanding the mechanism by which MMP-8 mediates this anti-tumour role is incomplete. In the breast, MMP-8 is expressed by the MEC population, and we identified loss of MMP-8 in DCIS-associated MECs [22]. This study aimed to address the role of MMP-8 in MECs and the impact of its loss on MEC phenotype and tumour-suppressor function.

We used purified cell populations from normal and diseased breast to confirm MECs as the primary source of MMP-8 in the normal breast, and its loss in DCIS-associated MECs. We demonstrated similar loss of expression in a cell line model of DCIS-associated MEC generated by over-expression of β6 integrin (β6-1089 cells) compared to its normal MEC counterpart (N-1089). These models were used to investigate gain-of-function effects, through over-expression of MMP-8 or an inactive mutant (EA, which carries a point mutation in catalytic site) in β6-1089, and loss-of-function effects, through MMP-8 knock-down in N-1089 cells.

In normal breast, MECs have a central role in preserving normal tissue architecture, [60], and correct MEC-matrix interactions are crucial for MECs to maintain cell polarity in the context of tumour suppression [52–54, 61]. We therefore aimed to investigate if MMP-8 is involved in ECM sensing and response.

When modified β6-1089 cells were seeded on different ECM components, WT MMP-8 but not the inactive MMP-8 EA resulted in a significant increase in MEC adhesion to ECM proteins including Fibronectin, Collagen-I, Laminin-I and Tenascin-C. Furthermore, transwell migration assays indicated that MEC migration towards Fibronectin, Collagen-I, Laminin-I and Tenascin-C was significantly downregulated only in MMP-8 WT cells, suggesting that MMP-8 contributes to stable anchorage of MECs to ECM, reflecting their role in normal breast. Both adhesion to and migration towards LAP, an established ligand of αvβ6 integrin [42], was consistently and significantly reduced, possibly reflecting a reduction in αvβ6 integrin activity. Similarly, knock-down of MMP-8 in N-1089 cells resulted in decreased adhesion and enhanced migration to ECM proteins, confirming a role for MMP-8 in matrix adhesion.

MEC attachment to basement membrane is achieved through HDs, which are stable adhesions critical for the integrity of epithelial cell monolayers. The integrin α6β4 nucleates HD formation through linkage to plectin and the intermediate filament cytoskeleton [43, 62]. Disassociation of HDs and localisation of α6β4 to actin-rich protrusions are characteristics of the acquisition of a migratory phenotype in basal cells, and has been well studied in migrating keratinocytes during wound healing [63–65]. Since loss of HD formation is recognised in DCIS-associated MEC [43], we sought to investigate the effect of MMP-8 on the subcellular localisation of α6β4. This revealed that, in MMP-8 WT over-expressing cells, there was significantly greater co-localisation of α6β4 to HD-associated plectin, with shorter and reduced number of retraction fibres, corresponding to a reduced migratory phenotype. Moreover, phalloidin staining of modified β6-1089 cells showed that MMP-8 WT cells significantly spread more on Fibronectin, in keeping with a more adhesive phenotype. In keeping with this, knock-down of MMP-8 in N-1089 cells resulted in significantly longer and increased number of retraction fibres compared to control cells, which we speculate indicates the acquisition of a more migratory phenotype.

Compromise of the MEC-basement membrane barrier is a key event in progression of DCIS to invasive cancer. Since MMP-8 impacts on MEC adhesion and HD formation, we sought to analyse whether altered MMP-8 expression by MEC influences their tumour-suppressor activity. Transwell invasion assays were used, in which MCF-7, MDA-MB-231 or SUM159 breast cancer cells were placed onto a Matrigel-coated porous membrane and allowed to invade towards MEC populations. A significant decrease in tumour invasion was observed for MDA MD 231 and SUM159 cells co-cultured with MMP-8 WT over-expressing β6-1089 compared to empty vector and MMP-8 EA, suggesting that MMP-8 contributes to MEC invasion-suppressor effect. This was further supported by enhanced transwell invasion for all tumour cells in the presence of N-1089 MMP-8 knock-down cells. A similar effect was detected in a more physiologically relevant 3D organotypic model reflecting the microenvironmental conditions of DCIS, incorporating a fibroblast populations as well as the MEC-tumour cell bilayer [41, 66].

Cooperation of tumour cells with the microenvironment is required to breach basement membrane [19, 62, 67]. Thus altered proteolytic activity of MECs could contribute to the invasive phenotype of breast cancer cells. We previously have shown that β6-1089 cells promote tumour invasion through TGF-β-mediated upregulation of MMP-9 [41]. A number of studies have indicated that MMP-8 can modulate TGF-β signalling [68, 69], and since we show that over-expression of MMP-8 WT in β6-1089 cells modifies their invasion-promoter effect, we investigated whether MMP-8 could influence TGF-β signalling and MMP-9 expression or activity in this cell population. This showed that phosphorylation of SMAD-2 was downregulated in β6-1089 cells expressing MMP-8 WT and enhanced in N-1089 cells following MMP-8 knock-down, supporting a role for MMP-8 in dampening TGF-β signalling. However, MMP-8 WT or EA did not have a significant effect on MMP-9 expression, though when modified β6-1089 cells were incubated on fluorescently labelled gelatin, the substrate of MMP-9, the area of degradation was significantly abrogated by MMP-8 WT cells. In contrast, gelatin degradation was significantly enhanced by knock-down in N-1089 cells, and importantly this effect could be rescued by MMP-9 inhibition or by MMP-9 knock-down. This suggests some form of inhibitory interaction between MMP-8 and MMP-9: it previously has been shown that MMP-8 can establish a complex with MMP-9 but the function of this complex remains elusive [30].

In tissues MECs of normal and benign ducts are consistently positive for MMP-8, whilst there is a significant progressive loss of this metalloproteinase through pure DCIS to DCIS with co-existing invasion (*p* = 0.001, Additional file 2: Table S2). This finding should be further validated on a larger cohort ideally with long-term follow-up.

## Conclusions

Overall these data indicate that MEC-derived MMP-8 acts as a tumour suppressor by promoting HD formation and enhancing cell adhesion to ECM, thus maintaining tissue architecture, and concomitantly downregulating breast cancer cell invasion in a proteolytic-dependent manner. MMP-8 appears to downregulate the TGF-β signalling evident in DCIS-associated MEC, and opposes MMP-9 activity, which we show promotes breast cancer cell invasion [27] and also has been shown to prevent HD formation [43].

Since both αvβ6 integrin [27] and MMP-8 are key regulators of myoepithelial pro- and anti-tumorigenic activity, these data support the development of a risk stratification profile that could be used clinically to tailor management of patients with DCIS. This is an approach currently being addressed by the LORIS trial [70].

## Additional files

Additional file 1: Table S1. Summary of breast tissue examined for MMP8. (DOC 27 kb)

Additional file 2: Table S2. Duct-by-duct analysis. (DOC 28 kb)

Additional file 3: Figure S1. Dual immunofluorescent staining of a normal breast duct showing MMP-8 (*green* Atlas, HPA02122,1:200) and p63 (*red* Abcam, Ab735, 1:50). The image shows predominant myoepithelial localisation of MMP-8. (TIF 7068 kb)

Additional file 4: Figure S2. Images of one representative organotypic gel fluorescently stained for myoepithelial marker p63 (*green*) (non-invading cell layer), nuclear marker DAPI (*blue*) and marker of invasive breast cancer cells Neso (*red*). From *left to right* images show gels comprising fibroblasts, MDA-MB-231 cells and MECs transfected with Empty Vector, MMP8 WT and MMP8 EA. The *final panel* shows a gel comprised of fibroblasts and MECs alone. Non-transfected MECs were used in the last panel. (TIF 986 kb)

Additional file 5: Figure S3. (i) Densitometry quantifying pSMAD2 versus tSMAD2 normalised to the loading control. MECs transfected with MMP-8 WT show a marked reduction of pSMAD2 compared to Empty Vector and MMP-8 EA at 5 minutes. (ii) Densitometry quantifying pSMAD2 versus tSMAD2 normalised to the loading control. MECs transfected with siRNA to MMP-8 demonstrated a markedly stronger pSMAD2 signal compared to control siRNA (siLUC). (TIF 336 kb)

## Abbreviations

CM: Conditioned media
Col-I: Collagen-I
Col-IV: Collagen-IV
DCIS: Ductal carcinoma in situ
EA: Inactive mutant
ECM: Extracellular matrix
Fib: Fibronectin
HD: Hemidesmosomes
IBC: Invasive breast cancer
Lam-I: Laminin-I
LAP: Latency-associated peptide
LEC: Luminal epithelial cell
MEC: Myoepithelial cell
MMP-8: Matrix metalloproteinase-8
SFM: Serum-free media
Ten-C: Tenascin-C
TGF-β: Transforming growth factor beta
WT: Wild-type
