# Supplementary material for: Loss of MMP-8 in ductal carcinoma in situ (DCIS)-associated myoepithelial cells contributes to tumour promotion through altered adhesive and proteolytic function
Source: Breast Cancer Res. 2017 Mar 23;19:33. doi: 10.1186/s13058-017-0822-9 (PMC5363009; doi:10.1186/s13058-017-0822-9)
Supplement: Supplementary file 1 — Summary of breast tissue examined for MMP8. (DOC 27 kb) [file 13058_2017_822_MOESM1_ESM.doc]

Table S1

|  | Cases | Summary |
| --- | --- | --- |
| Normal Cases | 7 | All positive, including Hyperplasia |
| Pure DCIS | 9 | None homogeneously positive, 5 heterogeneously positive; 4 negative |
| DCIS with Invasion | 9 | None homogenously positive, 1 heterogeneously positive; 8 negative |
