# Supplementary material for: Loss of MMP-8 in ductal carcinoma in situ (DCIS)-associated myoepithelial cells contributes to tumour promotion through altered adhesive and proteolytic function
Source: Breast Cancer Res. 2017 Mar 23;19:33. doi: 10.1186/s13058-017-0822-9 (PMC5363009; doi:10.1186/s13058-017-0822-9)
Supplement: Supplementary file 2 — Duct-by-duct analysis. (DOC 28 kb) [file 13058_2017_822_MOESM2_ESM.doc]

Table S2 – Duct by Duct analysis

|  |  | MMP8 |  |  |
| --- | --- | --- | --- | --- |
|  | Positive | Hetro | Negative | Total Ducts |
| Pure DCIS | 12 | 20 | 31 | 63 |
| DCIS + Invasion | 1 | 8 | 39 | 48 |
