# Supplementary figures and images for: Loss of MMP-8 in ductal carcinoma in situ (DCIS)-associated myoepithelial cells contributes to tumour promotion through altered adhesive and proteolytic function
Source: Breast Cancer Res. 2017 Mar 23;19:33. doi: 10.1186/s13058-017-0822-9 (PMC5363009; doi:10.1186/s13058-017-0822-9)

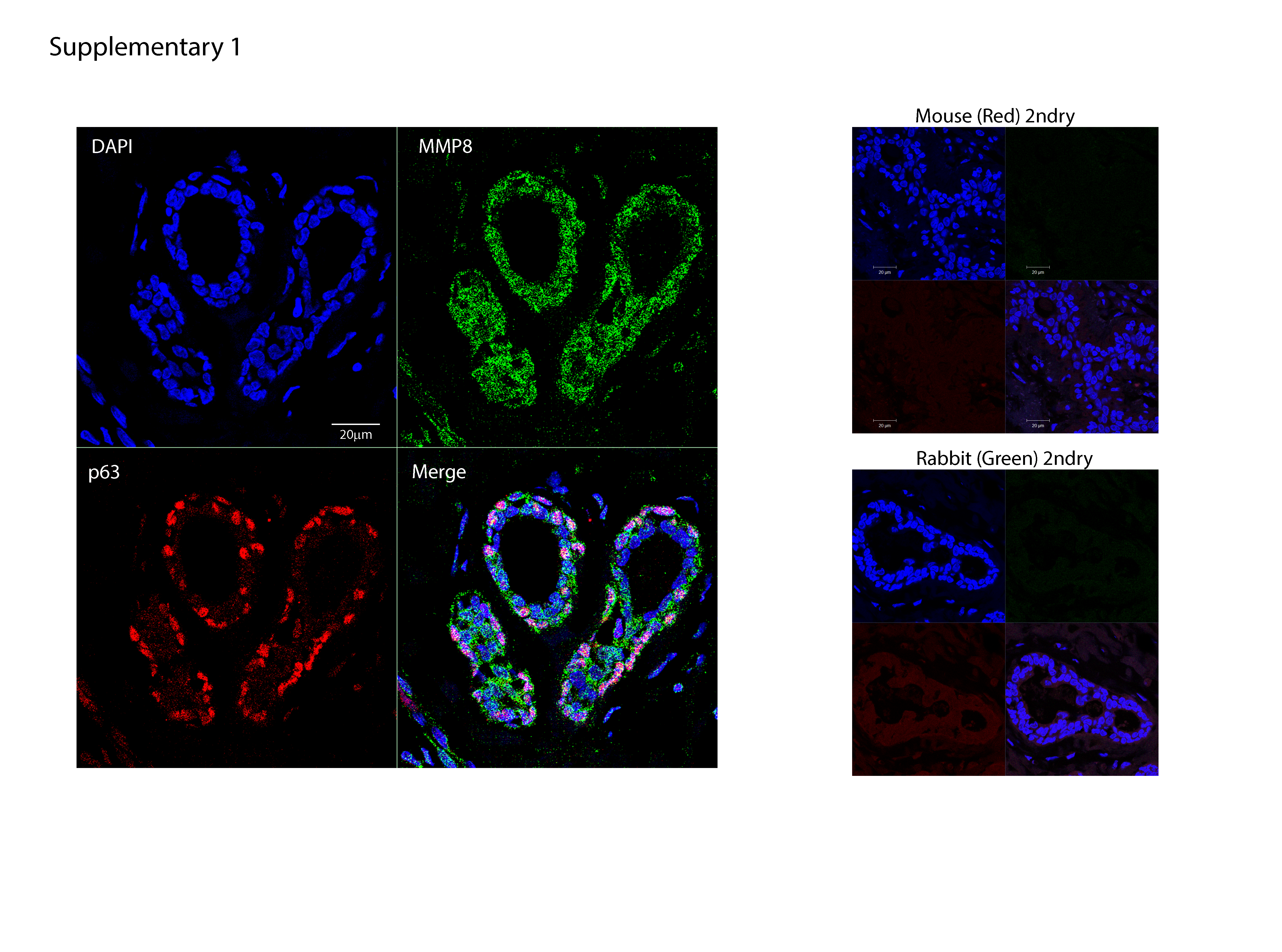

Supplement: Supplementary file 3 — Dual immunofluorescent staining of a normal breast duct showing MMP-8 (green Atlas, HPA02122,1:200) and p63 (red Abcam, Ab735, 1:50). The image shows predominant myoepithelial localisation of MMP-8. (TIF 7068 kb) [file 13058_2017_822_MOESM3_ESM.tif]

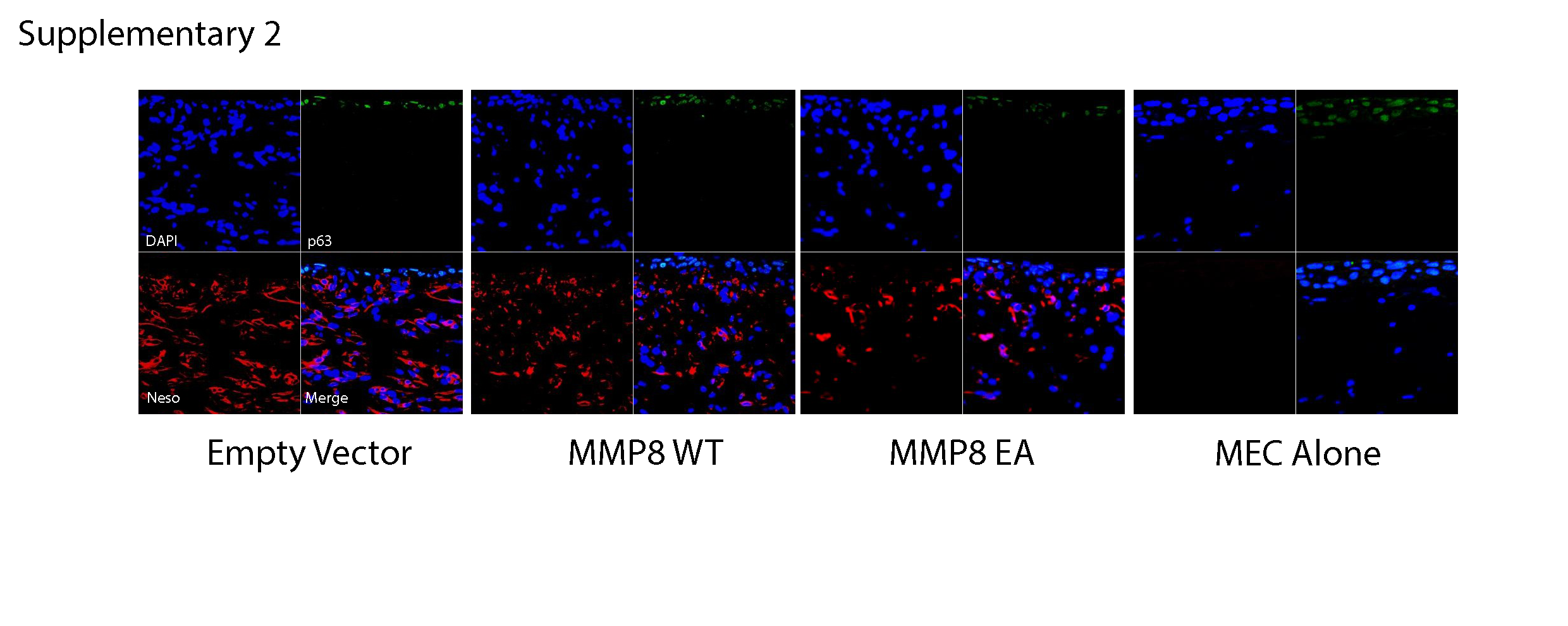

Supplement: Supplementary file 4 — Images of one representative organotypic gel fluorescently stained for myoepithelial marker p63 (green) (non-invading cell layer), nuclear marker DAPI (blue) and marker of invasive breast cancer cells Neso (red). From left to right images show gels comprising fibroblasts, MDA-MB-231 cells and MECs transfected with Empty Vector, MMP8 WT and MMP8 EA. The final panel shows a gel comprised of fibroblasts and MECs alone. Non-transfected MECs were used in the last panel. (TIF 986 kb) [file 13058_2017_822_MOESM4_ESM.tif]

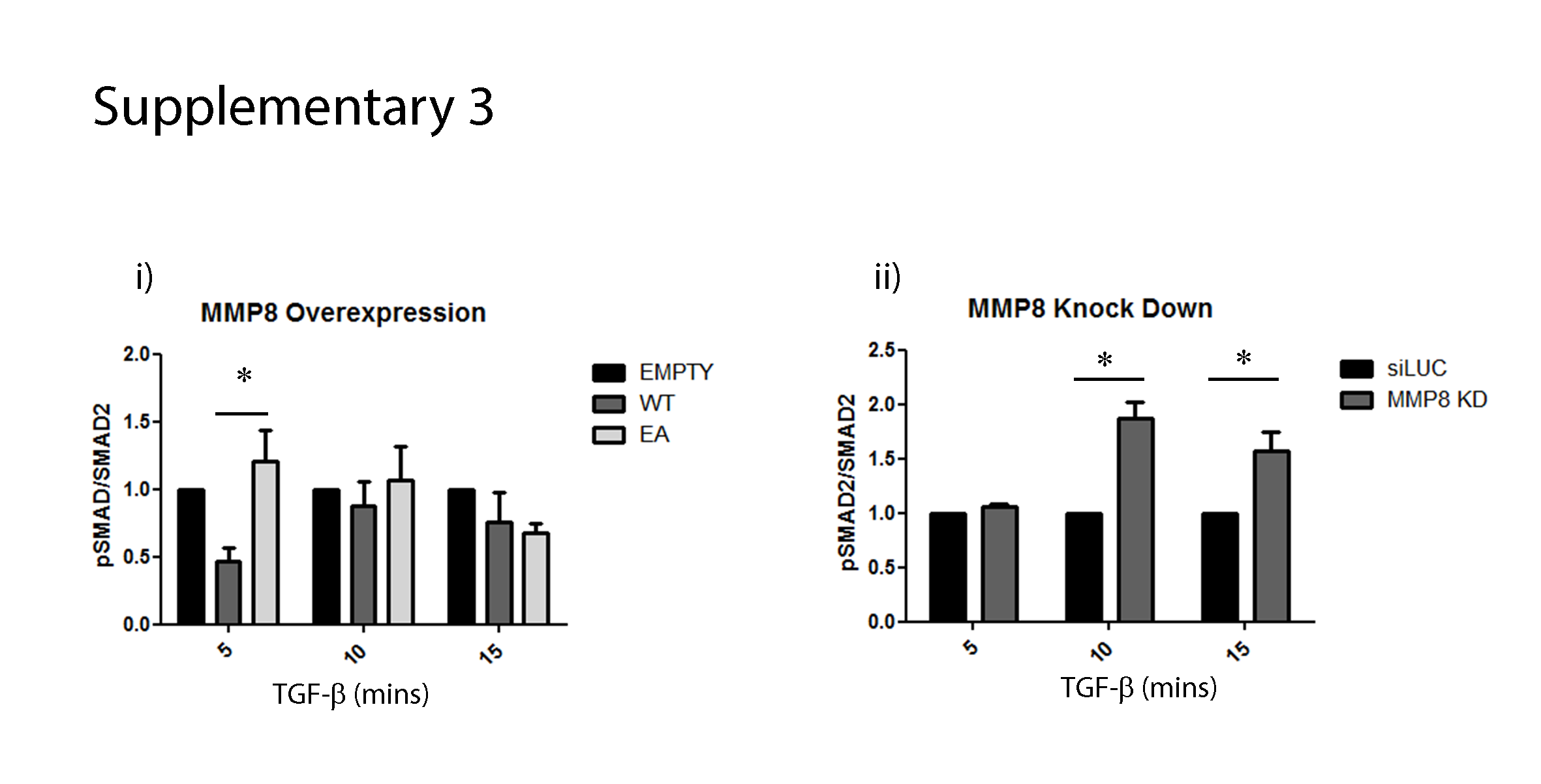

Supplement: Supplementary file 5 — (i) Densitometry quantifying pSMAD2 versus tSMAD2 normalised to the loading control. MECs transfected with MMP-8 WT show a marked reduction of pSMAD2 compared to Empty Vector and MMP-8 EA at 5 minutes. (ii) Densitometry quantifying pSMAD2 versus tSMAD2 normalised to the loading control. MECs transfected with siRNA to MMP-8 demonstrated a markedly stronger pSMAD2 signal compared to control siRNA (siLUC). (TIF 336 kb) [file 13058_2017_822_MOESM5_ESM.tif]
